# Biochemical characterization of patients with dihydrolipoamide dehydrogenase deficiency

**DOI:** 10.1002/jmd2.12382

**Published:** 2023-08-04

**Authors:** Parith Wongkittichote, Sanmati R. Cuddapah, Stephen R. Master, Dorothy K. Grange, Dennis Dietzen, Stephen M. Roper, Rebecca D. Ganetzky

**Affiliations:** ^1^ Division of Human Genetics Children's Hospital of Philadelphia Philadelphia Pennsylvania USA; ^2^ Department of Pathology and Laboratory Medicine Children's Hospital of Philadelphia Philadelphia Pennsylvania USA; ^3^ Division of Genetics and Genomic Medicine, Department of Pediatrics Washington University School of Medicine St. Louis Missouri USA; ^4^ Department of Pathology & Immunology Washington University School of Medicine St. Louis Missouri USA

**Keywords:** dihydrolipoamide dehydrogenase deficiency, lipoic acid, lysine degradation, mitochondrial disorder, urine organic acid analysis

## Abstract

Dihydrolipoamide dehydrogenase (DLD; E3) oxidizes lipoic acid. Restoring the oxidized state allows lipoic acid to act as a necessary electron sink for the four mitochondrial keto‐acid dehydrogenases: pyruvate dehydrogenase, alpha‐ketoglutarate dehydrogenase, branched‐chain α‐keto‐acid dehydrogenase, and 2‐oxoadipate dehydrogenase. DLD deficiency (DLDD) is caused by biallelic pathogenic variants in *DLD*. Three major forms have been described: encephalopathic, hepatic, and myopathic, although DLDD patients exhibit overlapping phenotypes. Hyperlactatemia, hyperexcretion of tricarboxylic acid cycle (TCA) metabolites and branched‐chain keto acids, increased plasma branched‐chain amino acids and allo‐isoleucine are intermittent metabolic abnormalities reported in patients with DLDD. However, the diagnostic performance of these metabolites has never been studied. Therefore, we sought to systematically evaluate the diagnostic utility of these biomarkers for DLDD. We retrospectively analyzed the results of biochemical testing of six unrelated DLDD patients, including values obtained during both well visits and acute decompensation episodes. Elevation of branched‐chain amino acid concentrations was not consistently observed. We found that five of six patients in our cohort had a maximum lifetime value of allo‐isoleucine of 6 μmol/L, showing that alloisoleucine elevations even during illness may be subtle. Urine organic acid analysis (UOA) during acute decompensation episodes was abnormal in all cases; however, the pattern of abnormalities had high intersubject variability. No single biomarker was universally present, even in patients experiencing metabolic decompensation. We also observed novel biochemical associations: three patients had hyperexcretion of TCA cycle metabolites during crisis; in two patients, 2‐ketoadipic and 2‐hydroxyadipic acids, by products of lysine degradation, were detected. We propose that these result from 2‐oxoadipate dehydrogenase deficiency, an underappreciated biochemical abnormality in DLD. Given the diversity of biochemical profiles among the patients with DLDD, we conclude that accurate biochemical diagnosis relies on a high index of suspicion and multipronged biochemical analysis, including both plasma amino acid and urine organic acid quantitation during decompensation. Biochemical diagnosis during the well state is challenging. We emphasize the critical importance of multiple simultaneous biochemical tests for diagnosis and monitoring of DLDD. We also highlight the under‐recognized role of DLD in the lysine degradation pathway. Larger cohorts of patients are needed to establish a correlation between the biochemical pattern and clinical outcomes, as well as a genotype–phenotype correlation.

## 1 INTRODUCTION

Dihydrolipoamide dehydrogenase (DLD, EC 1.8.1.4), also known as E3 subunit, is an enzyme encoded by the *DLD* gene (OMIM #238331) that oxidizes lipoic acid to regenerate its disulfide bond.^1^ This oxidation is critical to allow dihydrolipoamide to serve as an electron sink for the dehydrogenase activity of multiple enzyme complexes, including pyruvate dehydrogenase (PDH), alpha‐ketoglutarate dehydrogenase (KGDH), branched‐chain alpha‐keto acid dehydrogenase (BCKDH), and 2‐oxoadipate dehydrogenase (OADH).^2^, ^3^, ^4^ DLD is also involved in the glycine cleavage system, known as L‐protein.^5^

Pathogenic variants in *DLD* cause autosomal recessive DLD deficiency (DLDD, OMIM# 246900).^3^, ^6^ Three forms of DLDD have been described, although many patients exhibited overlapping features.^7^ The hepatic form is the most common, and is associated with an Ashkenazi Jewish founder variant: c.685G>T (p.Gly229Cys) with an estimated carrier frequency of 1:94 to 1:110 among the Ashkenazi Jewish population.^8^ The hepatic form of DLDD causes recurrent liver failure triggered by physical stress, fever, infection (especially Epstein Barr virus) and specific medications (ethanol, acetaminophen).^9^ The early‐onset neurologic form presents with vomiting, lethargy, hypotonia, developmental delay, seizures, and encephalopathy.^3^, ^10^ Some patients with the neurologic form may have Leigh syndrome.^11^, ^12^ Many patients with the hepatic form also exhibit neurologic involvement such as developmental delay or abnormal brain imaging, though not as severe as that seen in the early‐onset neurologic subtype.^13^, ^14^ The rarest form, reported in only two patients, is a late‐onset myopathic form.^15^, ^16^ Many individuals do not fit cleanly into one of these three categories.

Biochemical characteristics of patients with DLDD include elevated branched‐chain amino acids (BCAA), alanine, and allo‐isoleucine on the plasma amino acid profiles; and hyperexcretion of lactate, alpha‐ketoglutarate, TCA cycle intermediates and/or BCAA metabolites in urine organic acid analysis.^2^, ^7^, ^11^ However, it is known that patients with hepatic DLDD may not have biochemical profile abnormalities when they are well.^7^ The sensitivity and specificity of each individual analyte and of biochemical profiling overall are not known.

Here we analyzed biochemical profiles of patients with DLDD in our cohort. We emphasize that neither plasma amino acid nor urine organic acid analyses are 100% sensitive for diagnosis of DLDD, although some of biochemical markers may be useful for monitoring the metabolic status of patients with DLDD. We also identify relatively specific biochemical findings.

## 2 METHODS

We retrospectively analyzed the results of serial biochemical testing in 6 DLDD patients, including values obtained during both well visits and acute decompensation episodes. Patients were recruited between February 2015 and December 2022. Table 1 summarizes patient clinical and genotype data. All but one patient carried at least one copy of the recurrent c.685G>T variant (7/12 alleles). Clinical and biochemical information regarding Patient 6 was previously reported.^14^ Urine organic acid and plasma amino acid analyses were performed as previously described.^17^, ^18^

**TABLE 1:** Patient clinical characteristics and genotypes.

Patient	Gender	Age at initial evaluation	Clinical description	Genotype	Ethnic identity
1	F	22 years	Intermittent liver failure, normal developmental milestones	Homozygous c.685G>T (p.Gly229Cys)	Ashkenazi Jewish
2	M	15 months	Hypoglycemia, lactic acidosis, intermittent liver failure, developmental delay, epilepsy, abnormal brain imaging suggestive of metabolic stroke	c.685G>T (p.Gly229Cys) c.772T>C (p.Ser258Pro)	Mixed European, non‐Jewish
3	F	18 months	Recurrent lactic acidosis, hypoglycemia, intermittent liver failure, normal developmental milestones	c.685G>T (p.Gly229Cys) c.1046+5G>T	Ashkenazi Jewish
4	F	9 years	Intermittent liver failure, normal developmental milestones	Homozygous c.685G>T (p.Gly229Cys)	Ashkenazi Jewish
5	M	2 years	Developmental delay, Leigh syndrome, ataxia, exercise intolerance, metabolic stroke	Homozygous c.1123G>A (p.Glu375Lys)	Ashkenazi Jewish, Sephardi Jewish
6	F	3 years	Fulminant liver failure, developmental delay, Leigh syndrome, status epilepticus, status dystonicus, death due to respiratory failure	c.685G>T (p.Gly229Cys) c.548C>T (p.Thr183Met)	South American

## 3 RESULTS

The patient cohort includes a range of phenotypes of DLDD, including patients with acute/recurrent hepatic failure and/or encephalopathic episodes, Leigh syndrome, chronic encephalopathy and exercise intolerance. “Decompensations” include both episodes of acute hepatic dysfunction and episodes of acute encephalopathy. Elevated transaminases were seen (by definition) in all episodes of hepatic decompensation. Lactic acidosis was present in all decompensations, including both hepatic and encephalopathic episodes.

**FIGURE 1:**
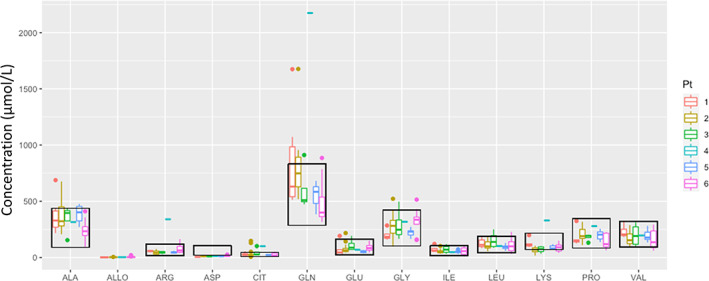
Plasma amino acid profiles of each patient during well and acute decompensation. Black boxes indicate normal range of each amino acids.

Plasma amino acid profiles of the patients with DLDD in our cohort were non‐specific overall (Figure 1). Alanine and glutamine were elevated in some patients during acute decompensation. Trace allo‐isoleucine was inconsistently detected, even during an episode of decompensation. Five out of the six patients (83%) had either normal or subtly elevated allo‐isoleucine (max 6 μmol/L; reference interval (RI) < 5 μmol/L). Patient 6 is the only individual in this cohort that had significantly elevated allo‐isoleucine to 21 μmol/L. Branched‐chain amino acids were mildly elevated or normal, including samples obtained during decompensation episodes. Citrulline was elevated at least intermittently in three patients (50%). Interestingly, low lysine was observed at least intermittently in 5 patients (83%), and in all but two samples. Although hypolysinemia has not previously been associated with DLD, in this cohort, it was the most sensitive amino acid marker.

**FIGURE 2:**
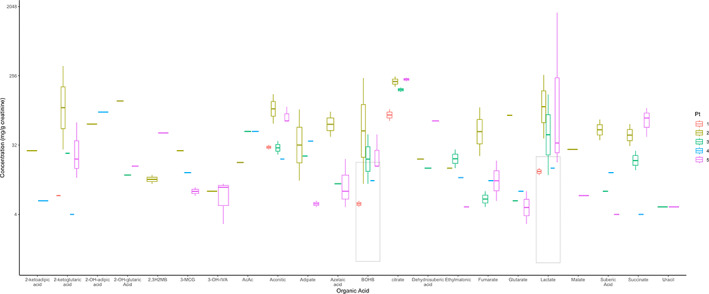
Urine organic acid profiles of each patient during well and acute decompensation. Gray boxes indicate normal range of each organic acids that are available. The amounts of organic acid are displayed in logarithmic scale.

Hyperexcretion of BCAA and TCA metabolites was detected in some patients during acute decompensation. However, the urine organic acid pattern was not consistent among patients, although it was relatively preserved across serial samples from any individual patient. The most common findings were non‐specific lactic aciduria and ketonuria. Increased fumarate and skewed succinate/fumarate ratio, in which fumarate level is higher than succinate, were detected in three patients during acute decompensation. These abnormalities normalized when the patients were clinically stable. Interestingly, 2‐ketoadipic and 2‐hydroxyadipic acids were detected in the urine of two individuals during acute decompensation and represent relatively specific markers of DLDD (Figure 2).

## 4 DISCUSSION

In this study, we performed serial biochemical analyses in six patients with DLDD. Defects in pyruvate metabolism, TCA cycle and BCAA catabolism are expected in patients with DLDD, leading to the accumulation of metabolites in those pathways.^7^ However, these findings were subtle and inconsistent, even during episodes of metabolic decompensation. Interestingly, each patient exhibited a distinct biochemical signature, reflecting an individualized metabolic perturbation. The only fully sensitive test for DLDD in this cohort was elevated blood lactate during decompensation episodes, which is obviously non‐specific. Similarly, elevated plasma alanine and glutamine and hyperexcretion of lactate on urine organic acid analysis were frequently seen but are nonspecific findings. Overall, we conclude that conventional biochemical testing does not provide an isolated or specific biomarker for diagnosing DLDD.

BCKDH is expected to be inhibited in DLDD; however, plasma amino acid analysis revealed normal or mildly elevated BCAAs, even during metabolic decompensation episodes. Only Patient 6 exhibited significant elevation of allo‐isoleucine during hepatic failure, while it was absent or only minimally elevated in the majority of the patients. Allo‐isoleucine is specific and highly sensitive for classic maple syrup urine disease (MSUD) caused by defects in BCKDH E1 and is used as a second‐tier biomarker for newborn screening.^19^ However, while it retains specificity it has low sensitivity to detect variant MSUD, including MSUD caused pathogenic variants in *DBT*, encoding BCKDH E2.^20^ Allo‐isoleucine has been undetectable in previous case reports of DLDD,^21^, ^22^, ^23^ which is consistent with our study. Citrulline was found to be elevated in 7/17 previously reported patients and was thought to be another potential marker for DLDD^24^, ^25^; however, citrulline was only ever elevated in 50% of our patients, and citrulline was low in some samples.

Urine organic acid analysis during metabolic decompensation revealed multiple metabolites including TCA metabolites (2‐ketoglutaric, 2‐hydroxyglutaric), BCAA metabolites (2‐ketoisocaproic, 2‐hydroxyisovaleric, and 2‐hydroxy‐3‐methylvaleric acids) and a skewed succinate/fumarate ratio. These findings support that urine organic acid analysis is a sensitive method for diagnosis and detection of decompensation episodes in DLDD patients; however, many of these findings are not specific. Abnormal biochemical findings normalized with the resolution of metabolic decompensation, which makes the diagnosis of the DLDD based on biochemical testing challenging.

Overall, these biochemical profiles also highlight the importance of DLD for lysine metabolism. Two of the patients exhibited 2‐ketoadipic and 2‐hydroxyadipic acids in urine. 2‐ketoadipic acid is a substrate for OADH, an enzyme involved with lysine, hydroxylysine and tryptophan catabolism.^26^ OADH is a thiamine‐dependent enzyme complex similar to other mitochondrial dehydrogenases.^26^, ^27^ Its E1 subunit is homologous to the KGDH E1 subunit.^26^ OADH shares its E2 subunit with KGDH and the E3 subunit with other mitochondrial dehydrogenases.^28^ Biallelic pathogenic variants in *DHTKD1*, encoding E1 subunit of OADH causes 2‐aminoadipic and 2‐ketoadipic aciduria (AAKAD, OMIM# 204750).^29^, ^30^ Given the role of E3 subunit in OADH, biochemical profiles of DLDD patients may overlap with AAKAD patients. It is unclear whether 2‐hydroxyadipic acid is formed by nonspecific enzymatic activity or reduction of 2‐ketoadipic acid during sample processing. This finding, which has been reported in other patients with suspected DLDD without molecular confirmation,^31^ has been reported previously in the patient with multiple mitochondrial dysfunctions syndrome due to defective *NFU1*,^32^ a protein involved in mitochondrial iron–sulfur cluster (Fe‐S) assembly. Given that lipoic acid synthase (LIAS) is an Fe‐S dependent enzyme, the defect in Fe‐S assembly causes dysfunction of LIAS and subsequently lipoic acid‐dependent enzyme. Therefore, 2‐ketoadipic and 2‐hydroxyadipic acids have been speculated to be potential biomarkers for DLDD, mitochondrial Fe‐S assembly defects and lipoic acid biosynthesis disorders.^5^, ^33^, ^34^ Although the sensitivity is relatively low in our cohort, elevation of lysine metabolites is relatively specific for DLDD, especially if seen in conjunction with other abnormalities. Interestingly, low plasma lysine levels were the most sensitive amino acid marker in this cohort of DLDD patients. In the process of lysine degradation, 2‐ketoglutarate is used in two steps: alpha‐aminoadipic semialdehyde synthase and alpha‐aminoadipate aminotransferase.^35^ Previously studies supported the impact of 2‐ketoglutarate availability on lysine degradation.^36^, ^37^ Excess 2‐ketoglutarate, due to disruption of KGDH in DLDD, may drive the conversion of lysine to 2‐ketoadipate via those two enzymes.

A recent study described patients with isolated KGDH deficiency (KGDHD) due to pathogenic variants in *OGDH*.^38^, ^39^ Clinical manifestations of KGDHD include developmental delay, epilepsy, abnormal movements and hypotonia, which partially overlap with DLDD. Biochemical analysis revealed lactic acidosis and hyperammonemia in association with intercurrent infections. Urine organic acid analysis in some patients showed lactic aciduria and hyperexcretion of TCA cycle metabolites, specifically 2‐ketoglutaric, 2‐hydroxyglutaric and fumaric acids; however, many of reported patients had a normal urine organic acid profile, and one patient had an abnormal profile that normalized after resolution of decompensation, similar to DLDD patients. Hyperexcretion of the BCAA metabolites, 2‐ketoadipic acid and 2‐hydroxyadipic acid, could be used to distinguish DLDD and KGDHD.

Interestingly, although DLDD patients exhibit variable biochemical profiles, even among patients with the same genotype, each individual tends to exhibit a consistent profile during their episodes of decompensation. Previous studies demonstrated heterogeneous underlying molecular mechanisms among *DLD* pathogenic variants, including disruption of dimerization, alteration of the binding affinity to corresponding enzyme complexes and reactive oxygen species generation.^4^, ^40^, ^41^ An example of the heterogeneity is demonstrated by p.Gly229Cys. Previous studies revealed that muscle biopsy from the patients with homozygous p.Gly229Cys demonstrated decreased E3 activity itself and E3‐dependent enzyme activities.^9^, ^42^ The residue Gly229 is located in NAD^+^‐binding site,^9^ therefore it was speculated that this variant may interrupt with cofactor binding. Recent studies showed that p.Gly229Cys may not have direct effect on specific E3 activity but instead increased reactive oxygen species (ROS) production, and may lead to oxidative stress, lipoic acid cofactor damage, and subsequent disruption of KGDH activity.^4^, ^43^ Given the various mechanisms, each pathogenic variant may impact E3‐containing enzyme complexes to different degrees, leading to biochemical diversity among the patients. It is also possible that additional variants in each component of the E3‐containing dehydrogenase complexes may play a role in the differential biochemical profiles among patients. Finally, these findings may be influenced by gene–gene interactions, dietary intake and/or microbiome profile.

Currently, treatment options for DLDD are limited. A patient with myopathic form has been reported to be response to riboflavin.^15^ However, fibroblasts derived from the patient with homozygous p.Gly229Cys did not respond to riboflavin supplementation.^9^ Many patients have been treated with protein restriction and vitamin supplementation, although the outcomes are inconclusive.^7^

One limitation of this study is that 5 out of 6 patients in our cohort had at least one copy of the c.685G>T allele. Among those, two patients were homozygotes. This allele, which is an Ashkenazi Jewish founder variant, is present in the majority of known cases. However, these findings may not be generalizable across other genotypes. Of note, the presence of the c.685G>T allele in non‐Jewish patients in this cohort and the presence of non‐founder alleles in Jewish patients in this cohort emphasize that DLD is a panethnic condition. While Ashkenazi patients may undergo targeted testing for the c.685G>T allele, our findings emphasize the importance of biochemical testing in patients with clinical signs of DLD as a complement to genetic testing to improve sensitivity. This study is also relatively small; however, DLDD is a rare disorder, and this is the largest study to date of serial biochemical laboratories in this patient population. Clinical, biochemical and molecular analyses of a larger cohort of DLDD patients are needed to establish genotype–phenotype and clinical‐biochemical phenotype correlations.

Overall, we demonstrate the challenges in biochemical diagnosis of DLDD and emphasize the critical importance of multiple simultaneous biochemical tests for diagnosis and monitoring of DLDD. Previously reported biochemical markers in DLDD have a lower sensitivity than previously believed, even at times of decompensation. We highlight the novel finding that the presence of lysine metabolites may prove relatively specific for DLDD. We also highlight the under‐recognized role of DLD in the lysine degradation pathway. Our study expands the understanding of the biochemical phenotype of DLDD.

## AUTHOR CONTRIBUTIONS

Parith Wongkittichote and Rebecca D. Ganetzky designed and conceptualized the study. Parith Wongkittichote, Rebecca D. Ganetzky, Sanmati R. Cuddapah, and Dorothy K. Grange performed clinical analysis of the patients. Parith Wongkittichote, Rebecca D. Ganetzky, Stephen R. Master, Stephen M. Roper, and Dennis Dietzen performed biochemical analysis of the patients. Stephen R. Master and Rebecca D. Ganetzky created figures. Parith Wongkittichote drafted the manuscript. All authors were involved with revising the manuscript. Rebecca D. Ganetzky supervised the study.

## FUNDING INFORMATION

Not applicable.

## CONFLICT OF INTEREST STATEMENT

The authors declare that they have no conflict of interest.

## ETHICS STATEMENT

No interventions performed on patients and no additional biological specimens collected from participants.

## ANIMAL RIGHTS

This article does not contain any studies with animal subjects performed by the any of the authors.

## INFORMED CONSENT

All procedures followed were in accordance with the ethical standards of the responsible committee on human experimentation (institutional and national) and with the Helsinki Declaration of 1975, as revised in 2000 (5).

## Data Availability

Data sharing is not applicable to this article as no new data were created or analyzed in this study.
